# Salivary Gland Scintigraphy in Patients with Sjogren’s Syndrome: A local Experience with Dual-tracer

**DOI:** 10.22038/aojnmb.2016.8029

**Published:** 2017

**Authors:** Wing Hang Luk, Jessie Tse Hang Yeung, Eliza Po Yan Fung, Chiu Ming Lok, Yuet Ming Ng

**Affiliations:** Department of Radiology, Princess Margaret Hospital, Hong Kong SAR

**Keywords:** Salivary gland Scintigraphy, Sjögren’s syndrome

## Abstract

**Objective(s):**

To review the findings of the patients with Sjögren’s syndrome (SS) having technetium99-m-pertechnetate (^99m^Tc-pertechnetate) and gallium67- citrate (Ga-67) salivary gland scintigraphy in the past eight years.

**Methods:**

The patients with SS, who were referred to our department for salivary gland scintigraphy during January -2008December 2015 were studied using both ^99m^Tc-pertechnetate and Ga-67 citrate scintigraphy.

**Results:**

Eighteen patients were included in the study, 17 of whom had positive findings on ^99m^Tc-pertechnetate salivary gland scintigraphy. One patient had negative parotid glands findings on ^99m^Tc-pertechnetate, but positive findings in Ga-67 study. Four patients had asymmetric involvement of the parotid glands, and one patient had asymmetric involvement of the submandibular glands in ^99m^Tc-pertechnetate salivary gland scintigraphy. On the other hand, one patient had only submandibular gland involvement in the ^99m^Tc-pertechnetate scan. Nine patients (18/9) had positive parotid gland findings on Ga-67 study. The involvements of the parotid glands were all symmetrical, except for one patient. No abnormal gallium uptake in the submandibular glands in our patients was noted.

**Conclusion:**

^99m^Tc-pertechnetate salivary gland scintigraphy is sufficient for the assessment in the majority of patients with SS. Ga-67 scintigraphy may be a useful supplementary test, especially if the result of ^99m^Tc-pertechnetate scintigraphy is not conclusive.

## Introduction

In our center, we adopted the American-European Consensus Group Criteria (revised international classification criteria) (^1^) for Sjogren’s syndrome (SS). This established criterion is a useful tool in clinical trials and epidemiological surveys, addressing the classification of SS both in primary and secondary entities. Due to addressed high sensitivity and specificity, this criterion is widely accepted in the clinical diagnosis of the disease (^2^).

Dry mouth is a common presentation in SS, and Technetium-99m-pertechnetate (^99m^Tc-pertechnetate) is usually used to evaluate the salivary gland function (^3^), and sequential salivary gland scintigraphy with salivary gland stimulation is applied to confirm lack of salivary secretion (^4^). However, few studies have evaluated the sensitivity and specificity of this scintigraphic study in SS. (^5^) On the other hand, gallium-67 citrate (Ga-67) is useful in evaluating the inflammatory status of the salivary glands (^6^). In our Nuclear Medicine Department, a protocol was developed with both tracers aiming to improve the detection of salivary gland involvement in SS patients. In this case series study, we reviewed our experience in the use of dual-tracer, ^99m^Tc-pertechnetate, and Ga-67 for the assessment of patients with SS in a single regional hospital during the past eight years. To the best of our knowledge, this is the first study dealing with the results of this dual-tracer in salivary gland scintigraphy for patients with SS.

## Methods

Patients who were referred to our department for dual-tracer (^99m^Tc-pertechnetate and Ga-67) salivary gland scintigraphy during January 2008-December 2015 and were confirmed with SS included in the study.

### Imaging technique

Studies conducted from 2008 to 2012 were performed with a single detector system (Philips Adac, Genesys), while Studies conducted after 2012 used a single-photon emission computed tomography imaging system (GE Healthcare, Discovery NM/CT 670). On day one, after intravenous injection of 10 mCi ^99m^Tc-pertechnetate, dynamic salivary gland scintigraphy was performed using a low energy, high resolution, and parallel-hole collimator. Dynamic images were collected into a 128×128 matrix with a 140 keV photopeak for ^99m^Tc-pertechnetate. Anterior sequential salivary gland images were obtained at 60 s per frame for 15 min. Fifteen min after the injection, stimulation by sialogogue (2 ml fresh lemon juice) was orally administered. Another set of anterior sequential salivary gland images was obtained at 60 s per frame for another 15 min, which was followed by static posterior and bilateral lateral views, and images were stored in 256×256 matrix.

After the ^99m^Tc-pertechnetate study, each patient received 4-5 mCi of Ga-67 intravenously, and 48 h delayed images were acquired using 85-325 keV window and appropriate collimation. Anterior, posterior, bilateral, and lateral head and neck images were obtained in a 256×256 matrix.

### Image review

**Figure 1:**
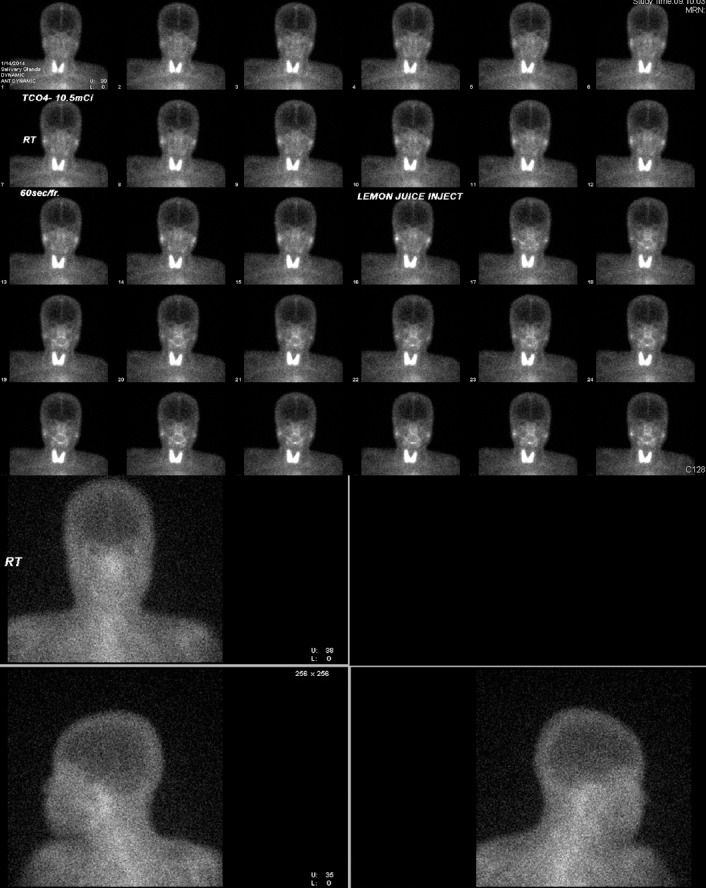
Images of a 49-year-old woman without Sjogren’s syndrome. (A) ^99m^Tc-pertechnetate scintigraphy shows good accumulation and good response for stimulation in both parotid and submandibular glands. (lemon juice at 15 min). (B) Gallium scan shows normal uptake in the bilateral parotid glands

The salivary glands were divided into four groups, namely, left parotid, left submandibular, right parotid, and right submandibular glands. ^99m^Tc-pertechnetate study was used for the assessment of accumulation of the radiotracer in the salivary glands and the secretory function after lemon juice stimulation. In a normal patient (Figure 1), a rapid uptake and increased concentration of the radioactive tracer were attained in the normal salivary glands within 10 min following intravenous administration of the radioactive tracer. After 15 min, the radioactive substance was rapidly secreted into the mouth after stimulation by diluted lemon juice. Abnormal scan was defined by salivary scintigraphy showing delayed uptake, reduced concentration, and/or delayed excretion of tracer according to the American-European Consensus Group Criteria (2).

In addition, the inflammatory status of these four groups of salivary glands was determined by the Ga-67 study with any increased uptake by the salivary glands. All the images were interpreted qualitatively and were reviewed by two experienced radiologists with special interest in nuclear medicine. Any discrepancy was resolved by consensus.

### Statistical analysis

The positive findings of the use of ^99m^Tc-pertechnetate and Ga-67 for salivary gland scintigraphy were documented using the clinical criteria for SS as the gold standard.

## Results

Eighteen patients with SS were included in the study and the results are summarized in Table 1. Seventeen patients were female; all the patients had positive antibody (Anti-Ro/SSA), most of them (11/18) had diagnosis of primary SS, and seven cases had secondary SS (six had systemic lupus erythematosus and one had rheumatoid arthritis). No observable significant difference was found between the primary and secondary SS in terms of salivary gland involvement by the two tracers.

**Table 1:** Clinical findings of patients with Sjogren’s Syndrome having dual-tracer scan

Patient no. ^Figure number^	Age	Sex	Lt Parotid		Rt parotid		Lt submandibular		Rt submandibular		Lt parotid	Rt parotid	Lt submandibular	Rt submandibular	Lacrimal gland	Duration of symptom (year)	Diagnosis

			Tc uptake	Tc secretion	Tc :uptake	Tc: secretion	Tc : secretion	Tc: secretion	Tc: uptake	Tc: secretion	Ga uptake						
1^2^	38	F	d	d	d	d	d	d	d	d	i	i	n	n	i	1	P
2^3^	40	F	n	n	n	n	d	d	d	d	i	i	n	n	i	2	S
3	71	F	d	d	d	d	d	d	d	d	n	n	n	n	n	2	S
4	43	F	n	n	n	n	n	n	n	n	i	i	n	n	i	1	S
5	82	F	d	d	d	d	d	d	d	d	i	i	n	n	n	5	P
6	53	F	d	d	d	d	d	d	d	d	n	n	n	n	n	2	S
7^4^	57	F	d	d	d	d	d	d	d	d	n	n	n	n	n	1	P
8^5^	60	F	d	n	d	n	d	d	d	d	n	n	n	n	n	1	P
9	36	F	d	d	d	d	d	d	d	d	n	n	n	n	n	2	P
10^6^	34	F	d	d	n	n	d	d	d	d	i	i	n	n	i	1	P
11	64	F	d	d	d	d	d	d	d	d	i	n	n	n	n	7	S
12	56	F	d	d	d	d	d	d	d	d	n	n	n	n	n	10	S
13	29	M	d	d	d	d	d	n	d	d	i	i	n	n	i	1	P
14	49	F	d	d	d	d	d	d	d	d	n	n	n	n	n	4	P
15	73	F	d	d	n	n	d	d	n	n	n	n	n	n	n	2	P
16	52	F	d	d	n	n	n	n	n	n	n	n	n	n	n	2	P
17	62	F	d	d	d	d	d	d	d	d	i	i	n	n	n	2	S
18	41	F	d	d	d	d	d	d	d	d	i	i	n	n	n	1	P

d: decrease i: increase n: normal P: Primary Sjogren’s Syndrome S: Secondary Sjogren’s Syndrome F: Female M: Male

Seventeen patients had positive findings on ^99m^Tc-pertechnetate salivary gland scintigraphy. Four patients (patients 8, 10, 15, and 16) had asymmetric involvement of the parotid glands, and one patient (patient 13) had asymmetric involvement of the submandibular glands in ^99m^Tc-pertechnetate salivary gland scintigraphy. Furthermore, one patient (patient 2) only had submandibular gland involvement in the ^99m^Tc-pertechnetate scan.

Nine patients (9/18) had positive parotid gland findings on Ga-67 study, and five of these patients showed increased lacrimal gland uptake, as well. One patient showed negative parotid gland findings on ^99m^Tc-pertechnetate, but positive findings in the Ga-67 study (patient 4). The durations of symptoms for negative and positive Ga-67 scan were 4.1 and 2.3 years, respectively. The involvements of the parotid glands were all symmetrical, except for one patient (patient 11). In our patients, no abnormal Ga-67 uptake was noted in the submandibular glands.

## Discussion

Primary SS occurs in the absence of other underlying autoimmune disorders, whereas secondary SS is associated with another underlying autoimmune disease, such as systemic lupus erythematosus, rheumatoid arthritis, or scleroderma. Radionuclide imaging can be employed to assess the involvement of salivary glands, especially in patients with xerostomia symptom. ^99m^Tc-pertechnetate is the most commonly used radioactive pharmaceutical agent, which roughly correlates with salivary gland biopsy findings, and it is a direct test of secretory function (^7^). In this test, the isotope is concentrated and excreted by the salivary glands, which allows demonstration of uptake in the salivary glands.

Sequential salivary scintigraphy with ^99m^Tc-pertechnetate confirms lack of salivary secretion and xerostomia. Nevertheless, decreased or absent ^99m^Tc-pertechnetate uptake by the salivary glands is a nonspecific phenomenon. Unilateral decreased uptake of ^99m^Tc-pertechnetate has been reported in patients with primary metastatic tumors, congenital aplasia, radiation sialadenitis, obstructive sialolithiasis, and surgical ablation; bilaterally diminished uptake may be observed in patients with other systemic connective-tissue diseases, acute suppurative parotiditis, multi-centric sialoangiectasis, and physiologic aging, as well as in those with SS (^8^). Yet, it should be possible to distinguish primary acinar ductal failure, as in SS, from both obstructive disorders and primary denervation states in which the glands may concentrate ^99m^Tc-pertechnetate, but not discharge it properly (8). Therefore, abnormal finding in salivary scintigraphy should not be considered pathognomonic of SS and other clinical criteria for diagnosis of SS are mandatory.

In practice, ^99m^Tc-pertechnetate salivary gland scintigraphy could be interpreted by direct visualization (7) or semi-quantitative (^9^, ^10^). We used simple direct visual analysis of salivary gland scintigraphy because it showed greater diagnostic utility in the diagnosis of SS (^11^).

Ga-67 is frequently used for studying inflammatory or neoplastic disease of the salivary glands. Ga-67 is taken up by dividing cells, and excessive gallium accumulation is observed in inflammatory process (^12^). However, inflammatory destruction of the salivary and lacrimal glands may not fully account for the symptoms of SS (^13^). There have been scattered reports of Ga-67 uptake in the lacrimal and salivary glands of patients with SS (^14^-^16^). Ga-67 scintigraphy may also be useful in the evaluation of ocular inflammatory status in patients with SS.

**Figure 2:**
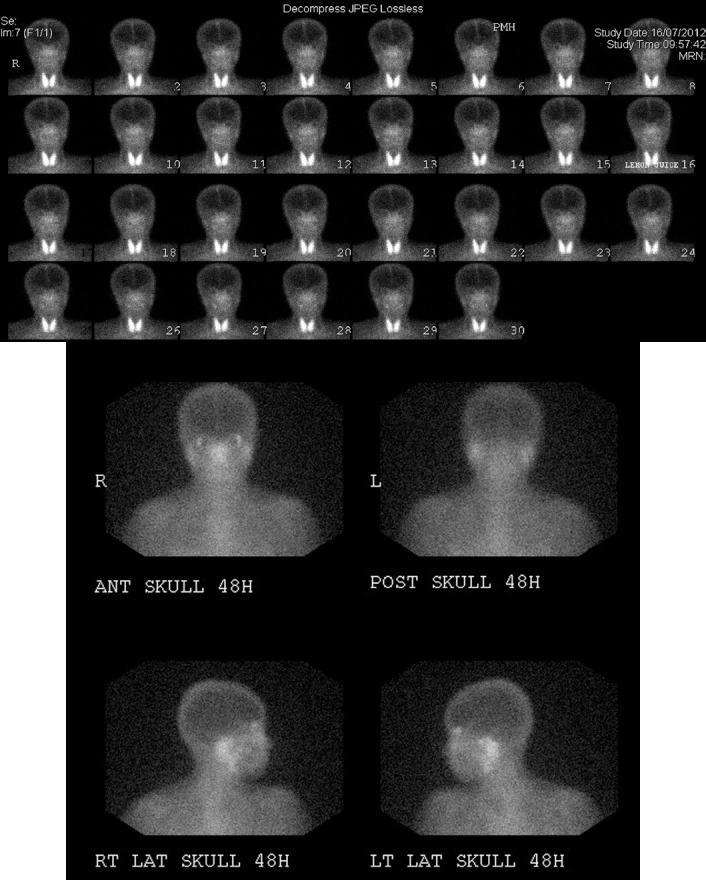
Images of a 38-year-old woman (Patient 1). (A) ^99m^Tc-pertechnetate scintigraphy shows minimal accumulation of tracer in submandibular glands and parotid glands. (B) Gallium scan illustrates moderatly increased uptake in the bilateral parotid glands and the lacrimal glands

**Figure 3:**
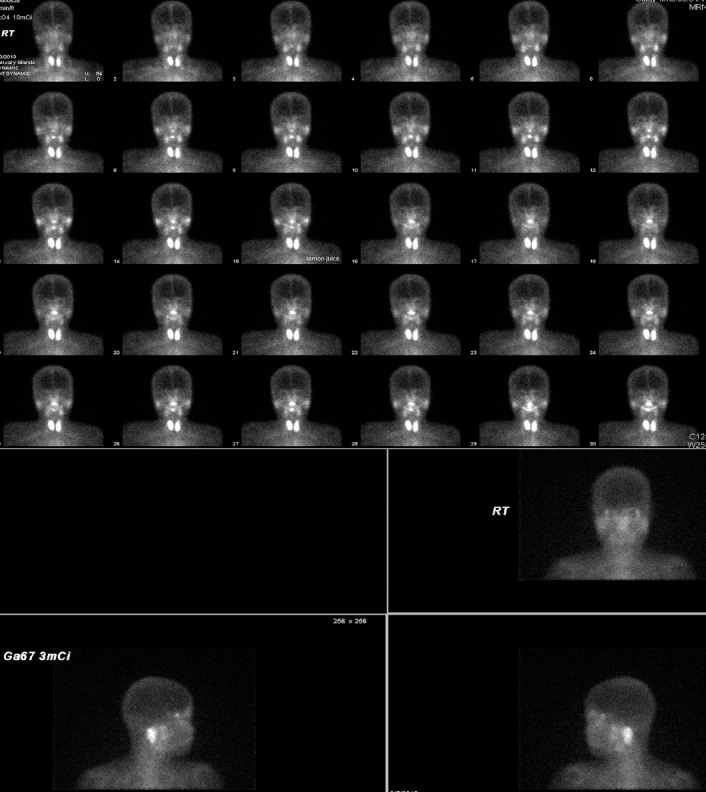
Images of a 43-year-old woman (Patient 4). (A) ^99m^Tc-pertechnetate scintigraphy exhibits normal accumulation of tracer in both parotid and submandibular glands with normal excretory function after stimulation (lemon juice at 15 min). (B) Gallium scan shows moderate to marked increased uptake in the bilateral parotid glands and mild increased uptake in the lacrimal glands

**Figure 4:**
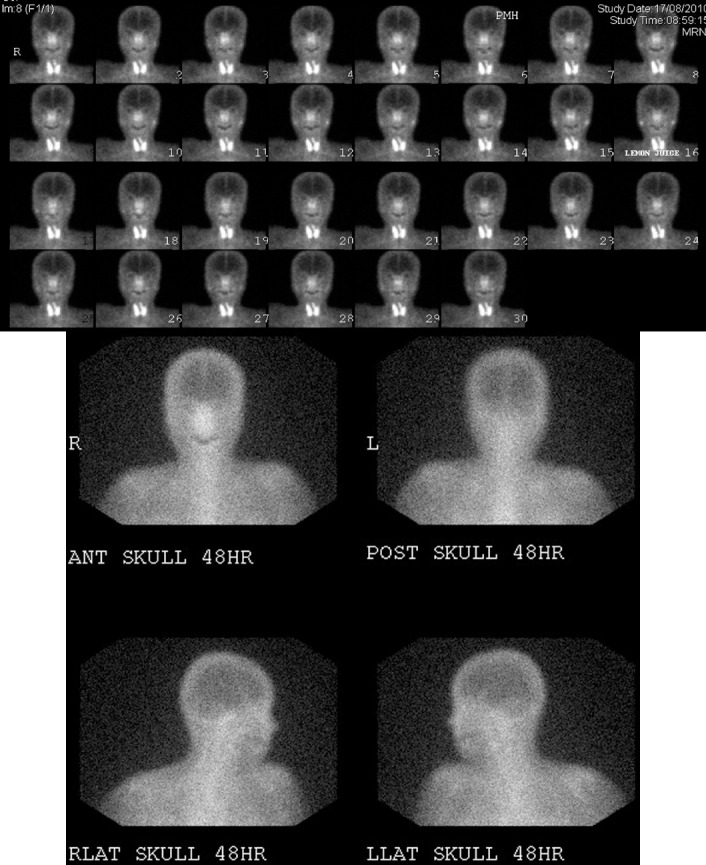
Images of a 57-year-old woman (Patient 7). (A) ^99m^Tc-pertechnetate scintigraphy shows marked decreased accumulation of tracer in submandibular glands and parotid glands. (B) Gallium scan of the bilateral parotid glands is normal

**Figure 5:**
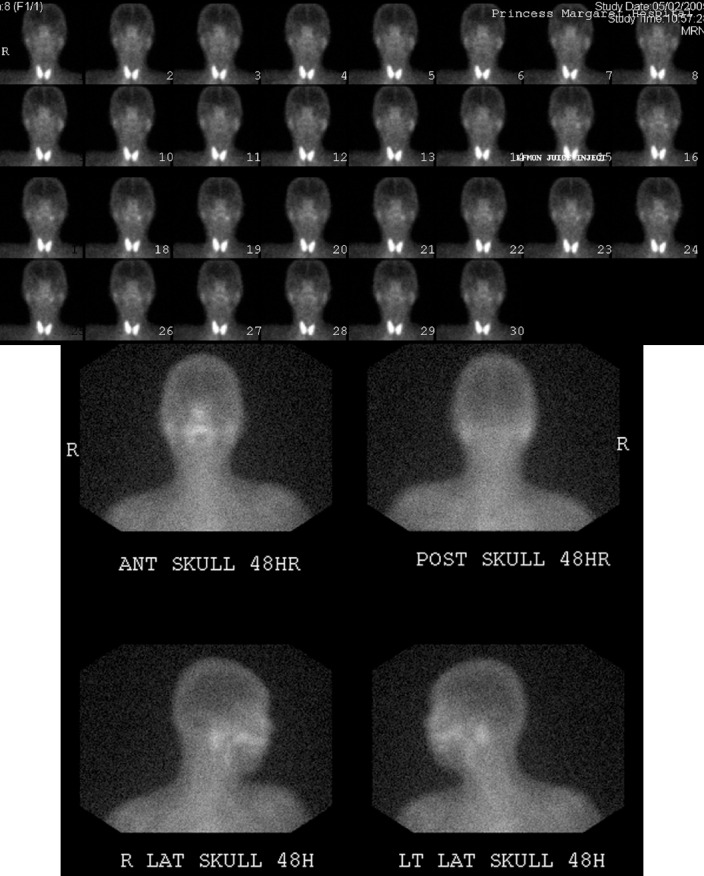
Images of a 34-year-old woman (Patient 10). (A) ^99m^Tc-pertechnetate scintigraphy exhibits decreased accumulation of tracer in left parotid gland. (B) Gallium scan shows marked increased uptake in the bilateral parotid glands and mildly enhanced uptake in the lacrimal glands

**Figure 6:**
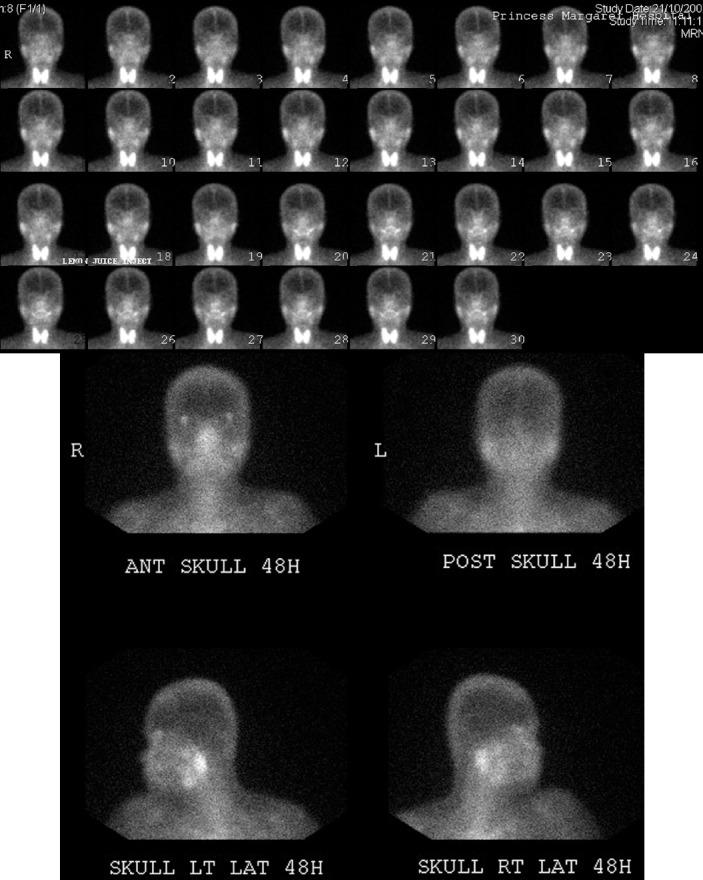
Images of a 60-year-old woman (Patient 11). (A) ^99m^Tc-pertechnetate scintigraphy shows decreased and minimal accumulation of tracer in parotid glands and submandibular glands, respectively. Both parotid glands show normal excretory function after stimulation (lemon juice at 15 minutes). (B) Gallium scan shows normal uptake in the bilateral parotid glands. No abnormal uptake is noted in the lacrimal gland

The results of our study showed heterogeneous presentations in these 18 patients (Figures 2-6). For instance, one patient (patient 2) only had submandibular gland involvement in the ^99m^Tc-pertechnetate scan. Aung Y et al. (^17^) suggested that the function of the submandibular gland was more affected than that of the parotid glands. However, no positive Ga-67 scan in the submandibular glands was noted in our study.

In addition, although SS is a systemic disease, there is heterogeneity of the salivary gland involvement, which may be due to the underlying disease severity. Güne S et al. proposed that asymmetric activity pattern in the parotid glands is common in early stage of the disease and it may be a predictor of progression (^18^). Similarly, our study showed that the duration of the symptoms in our patients with asymmetric involvement was one to two years.

All the patients, except for one, had changes in ^99m^Tc-pertechnetate scan, but only half of the patients had abnormal findings in Ga-67 scan. This might be explained by the fact that most patients in the study were having symptoms for more than one year; thus, the secretory function of the salivary glands was already damaged. The active inflammatory process of the salivary glands might have been subsided before the Ga-67 scan. Nonetheless, one patient (patient 4) had abnormal Ga-67 result, but normal ^99m^Tc-pertechnetate finding; Therefore, the use of gallium scan can be used as a supplement to ^99m^Tc-pertechnetate study to increase sensitivity.

The limitations of our study were its retrospective design and lack of salivary gland biopsy result, although all the patients fulfilled the diagnostic criteria. Given the small sample size, our study may serve as a pilot, and further prospective studies may be helpful.

## Conclusion

^99m^Tc-pertechnetate sialoscintigraphy is sufficient for the assessment of the status of the salivary glands in the majority of patients with SS. The use of Ga-67 scan may be a useful as a supplementary test, especially if the result of ^99m^Tc-pertechnetate scintigraphy is inconclusive.
